# Single-Step Formation of Ni Nanoparticle-Modified Graphene–Diamond Hybrid Electrodes for Electrochemical Glucose Detection

**DOI:** 10.3390/s19132979

**Published:** 2019-07-05

**Authors:** Naiyuan Cui, Pei Guo, Qilong Yuan, Chen Ye, Mingyang Yang, Minghui Yang, Kuan W. A. Chee, Fei Wang, Li Fu, Qiuping Wei, Cheng-Te Lin, Jingyao Gao

**Affiliations:** 1 MOE Key Laboratory for Non-Equilibrium Synthesis and Modulation of Condensed Matter, Xi’an Jiaotong University, Xi’an 710049, China; 2 Key Laboratory of Marine Materials and Related Technologies, Zhejiang Key Laboratory of Marine Materials and Protective Technologies, Ningbo Institute of Materials Technology and Engineering (NIMTE), Chinese Academy of Sciences, Ningbo 315201, China; 3 Department of Physics, Liaoning University, Shenyang 110000, China; 4 Department of Electrical and Electronic Engineering, Faculty of Science and Engineering, University of Nottingham, Ningbo 315100, China; 5 College of Material Science and Optoelectronic Technology, University of Chinese Academy of Sciences, 19 A Yuquan Rd., Shijingshan District, Beijing 100049, China; 6 Ningbo Institute of Materials Technology and Engineering (NIMTE), Chinese Academy of Sciences, Ningbo 315201, China; 7 Laser Research Institute, Shandong Academy of Sciences, Qingdao 226100, China; 8 College of Materials and Environmental Engineering, Hangzhou Dianzi University, Hangzhou 310018, China; 9 School of Materials Science and Engineering, Central South University, Changsha 410083, China

**Keywords:** glucose, electrochemical detection, Ni nanoparticles, graphene–diamond hybrid electrodes, sp^3^-to-sp^2^ conversion

## Abstract

The development of accurate, reliable devices for glucose detection has drawn much attention from the scientific community over the past few years. Here, we report a single-step method to fabricate Ni nanoparticle-modified graphene–diamond hybrid electrodes via a catalytic thermal treatment, by which the graphene layers are directly grown on the diamond surface using Ni thin film as a catalyst, meanwhile, Ni nanoparticles are formed in situ on the graphene surface due to dewetting behavior. The good interface between the Ni nanoparticles and the graphene guarantees efficient charge transfer during electrochemical detection. The fabricated electrodes exhibit good glucose sensing performance with a low detection limit of 2 μM and a linear detection range between 2 μM–1 mM. In addition, this sensor shows great selectivity, suggesting potential applications for sensitive and accurate monitoring of glucose in human blood.

## 1. Introduction

Diabetes, caused by the improper secretion of insulin, is one of the most serious and prevalent metabolic diseases in human beings. In order to monitor blood insulin levels, a reliable and accurate technique for the detection of blood glucose concentration is of importance [1,2,3,4]. To date, there have been many sensing approaches, for example, electrochemical [5], spectrophotometric [5], surface plasma resonance [6], and fluorescence methods [7], which are effective for glucose detection. Among these techniques, electrochemical glucose sensors have particular advantages, including process simplicity, high sensitivity, good selectivity, and low production costs. Generally, electrochemical sensors applied for the recognition of glucose oxidase can be classified as two types: enzymatic and non-enzymatic sensors [8,9]. Enzymatic glucose sensors may be easily influenced by external environmental conditions, such as temperature, pH value, humidity etc. [10,11], therefore non-enzymatic glucose sensors have drawn more attention from the scientific community over the past few years.

In recent years, great efforts have been devoted to exploring pragmatic and reliable non-enzymatic glucose sensors [12,13,14,15]. Until now, nanomaterials including metals and their oxides (such as Au, Ag, Co, Cu, Ni, Co_3_O_4_, NiCoO_2_, and NiO), as well as alloy systems (Ni–Pd, Cu–Pd, Ni–Cr etc.), have been developed as electrode materials for non-enzymatic glucose detection [16,17,18,19]. Among these nanomaterials, nickel and its derivatives have drawn great attention due to their non-toxicity, low cost, and excellent electrocatalytic activity for glucose oxidation, as NiOOH is an outstanding oxidizing agent in alkaline medium [20]. Moreover, according to Toghill’s work [20], nickel has been demonstrated to be the most sensitive electrode material applied for recognition of non-enzymatic glucose. Therefore, many non-enzymatic glucose sensors have been fabricated by decorating the substrate electrode with Ni nanoparticles (Ni NPs) or nanocomposites. You et al. prepared a glucose sensing electrode by dispersion of Ni NPs inside graphite-like carbon [21], and Safavi et al. mixed nano-sized nickel hydroxide with ionic liquid and graphite powder to fabricate a composite electrode for detecting glucose [22]. However, the simple combination of Ni nanomaterials and the substrate electrode still has a problem with long-term electrochemical stability in the electrolyte environment, owing to the weak interfacial interaction (van der Waals forces) between them. In order to solve this issue, the development of Ni NPs-modified electrochemical electrodes with a strong covalent binding at their interface is of significance for glucose detection [12,23,24,25].

Graphene, one of the attractive two-dimensional materials, has drawn enormous attention owing to its unique physical properties, for instance, high carrier mobility (≈15,000 cm^2^/V·s), good chemical stability, and high specific surface area (≈2600 m^2^/g) [26,27]. Graphene-based electrochemical sensors have experienced extensive application in electroanalysis and enabled the development of biosensors with high sensitivity and specificity [28,29,30]. In common cases, such sensors were fabricated by depositing graphene sheets upon the surface of a glassy carbon electrode (GCE), followed by the decoration of Ni NPs on the graphene surface [31,32]. Again, this sensing platform has the same problem of weak adhesion at the interface of Ni NPs/graphene and graphene/GCE through van der Waals interactions [33]. 

In this contribution, we developed a single-step method to fabricate Ni NPs-modified graphene–diamond hybrid electrodes via a catalytic thermal treatment process. A sp^3^-to-sp^2^ conversion method was proposed in our previous study to directly grow few-layer graphene on the surface of diamond, using metal film as a catalyst and diamond itself as a carbon source [28,30]. In this study, during catalytic annealing for graphene growth, Ni NPs could be simultaneously formed on the surface of graphene–diamond hybrid electrodes due to the aggregation of Ni thin films into Ni NPs based on the dewetting behavior. The fabricated glucose sensors have a low detection limit of 2 μM with a linear electrochemical detection range from 2 μM to 1 mM. The better sensing performance is credited to the enhanced electrocatalytic activity of Ni NPs and the covalent bonding between Ni NPs and graphene. 

## 2. Materials and Methods 

We first purchased single-crystal high-pressure and high-temperature (HPHT) diamonds with a size of 3.5 × 3.5 × 1 mm and (1 0 0) orientation from the Shenzhen Tiantian Xiangshang Diamond Co. Ltd., Shenzhen, China. In order to remove the surface contaminants from the diamonds, we immersed diamond substrate into a piranha solution composed of 70% H_2_SO_4_ and 30% H_2_O_2_ in volume (purchased from the Sinopharm Chemical Reagent Co. Ltd., Shanghai, China) at 50 °C for 4 h. The treated diamonds were further cleaned in deionized water and ethanol for 10 min, respectively, by ultrasonic cleaning. The clean diamonds were then deposited with a 50 nm-thick Ni film on their surface via an e-beam system under the pressure of 1.4 × 10^−5^ Torr, and a deposition rate of 0.5 Å/s (MUE-ECO, Chigasaki, Japan). After the deposition process, we placed the Ni/diamond substrate in a tube furnace system, followed by thermally treating them in 8 sccm H_2_ flow at 1020 °C for 15 min (BTF-1200C-II-SL, Hefei, China). We finally fabricated a device for the electrochemical experiment by immobilizing the as-prepared sample on a plastic substrate with silver wire connected to it through silver paint. The other exposed area was protected with silicone resin. 

Some characterization methods were applied to investigate our samples. We first determined the chemical composition change to the diamonds before and after thermal treatment using X-ray photoelectron spectroscopy (XPS AXIS ULTR DLD, Kratos Analytical, Manchester, UK). Following that, we used Raman spectroscopy (Renishaw inVia Reflex, Renishaw plc, Wotton-under-Edge, UK) to characterize the quality of the diamonds and obtained graphene. A field emission scanning electron microscope (FE-SEM QUANTA 250 FEG, FEI, Hillsboro, OR, USA) was then applied to observe the surface morphology of the samples. Finally, we conducted some electrochemical experiments with an Autolab workstation (PGSTAT 302F, Metrohm, Herisau, Switzerland) to investigate the electrochemical performance of our device. 

## 3. Results and Discussion

**Figure 1:**
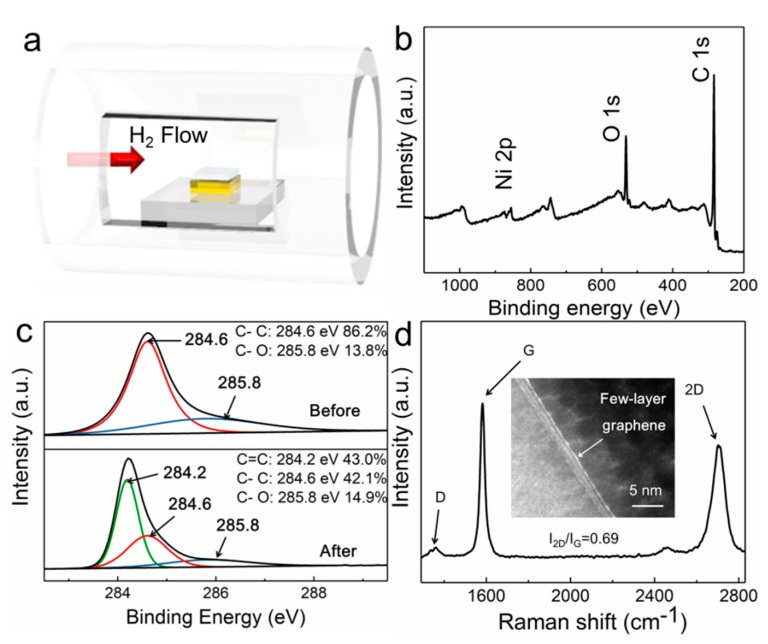
(**a**) The preparation process of Ni NPs-modified graphene–diamond hybrid electrodes. (**b**) X-ray photoelectron spectroscopy (XPS) survey scan and (**c**) high-resolution C1s spectra of the diamond surface before and after the catalytic annealing process. (**d**) Raman spectrum of the obtained composite electrodes. Inset of (**d**) TEM image of the hybrid electrode.

Our proposed process for the fabrication of Ni NPs-modified graphene–diamond hybrid electrodes based on sp^3^-to-sp^2^ conversion during catalytic annealing is illustrated in Figure 1a. In brief, a piece of HPHT diamond was pre-deposited with a 50 nm-thick Ni thin film, followed by setting it in a tube furnace system with H_2_ flow. After annealing at 1020 °C for 15 min, the system was naturally cooled down. The chemical components of the surface of the diamond before and after the catalytic annealing process were characterized by XPS analysis. As shown in Figure 1b, after annealing, the survey scan indicated that the sample surface contained Ni, O, and C signals. Additionally, in Figure 1c, the surface of pristine diamond was composed of complete sp^3^ C–C bonds except for some oxygen-containing groups [34]. In contrast, the ratio between the sp^2^ and sp^3^ bonds of the sample increased equal to 1:1 after the sp^3^-to-sp^2^ conversion process. The quality of the in situ formed graphene was examined by Raman spectroscopy. As presented in Figure 1d, the typical peaks located at 1351, 1586, and 2699 cm^−1^ can be assigned to the D-peak, G-peak, and 2D-peak of graphene, respectively [35]. The narrow (≈70) full width at half maximum (FWHM) of the 2D-peak and the I_2D_/I_G_ ratio (≈0.69) suggests the characteristics of few-layer graphene, while the TEM image shown in the inset of Figure 1d indicates that few-layer graphene was directly formed on the diamond surface after catalytic thermal treatment. Moreover, the low I_D_/I_G_ ratio (<0.1) indicates that the graphene transformed from the diamond has low defect content [36]. The graphene layers played a role as electrical conducting films, and the good interface between the Ni nanoparticles and the graphene guaranteed an efficient charge transfer during electrochemical detection.

**Figure 2:**
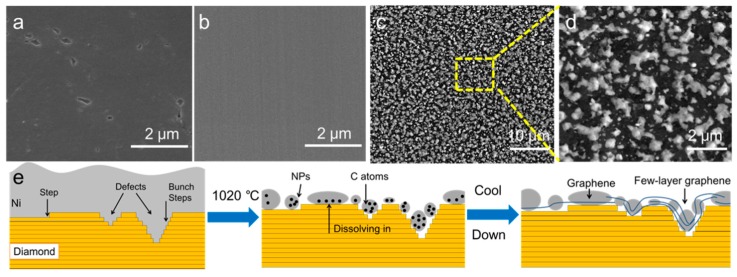
SEM images showing the surface morphology of (**a**) high-pressure and high-temperature (HPHT) diamond; (**b**) Ni thin film-coated diamond; (**c**,**d**) Ni NPs-modified graphene–diamond hybrid. (**e**) The mechanism for single-step formation of Ni NPs-modified graphene–diamond hybrid electrodes.

Figure 2a–d presents a comparison of the surface morphology between the HPHT diamond, the Ni thin film-coated diamond, and the Ni NPs-modified graphene–diamond hybrid, respectively. In Figure 2a,b, both the pristine and 50 nm-thick Ni film-coated diamond substrates exhibit a smooth and flat surface with some void defects. After thermal treatment at 1020 °C, the sample surface becomes coarse and a high density of uniformly distributed nanoparticles are formed, as shown in Figure 2c,d. The EDS analysis was performed to acquire the elemental content of these nanoparticles, and only C and Ni peaks can be seen, corresponding to the observation from the XPS survey plot. Thus, we conclude that these nanoparticles are composed of Ni, and as an example, the Ni NPs has a diameter of ≈178 nm. The mechanism for single-step formation of Ni NPs-modified graphene–diamond hybrid is proposed as an anisotropic etching process, as illustrated in Figure 2e. It can be explained that at high temperature (1020 °C), the carbon atoms may diffuse and dissolve into Ni thin film and this solid–solution reaction proceeds laterally along the steps on the surface of the diamond. As there have been some void defects incorporated in and on the HPHT diamond, Ni etching behavior becomes more severe at the defect region, leading to the promotion of solid–solution reaction [37]. Meanwhile, Ni thin film would gradually aggregate into Ni NPs with various sizes due to the dewetting phenomenon at high temperature [38]. During the cooling process, the supersaturated carbon atoms in the Ni NPs would precipitate to form graphene layers through an out-diffusion process [39]. As a result, a Ni NPs-modified graphene–diamond hybrid may have a larger specific active surface area and better electrocatalytic activity for further electrochemical detection.

**Figure 3:**
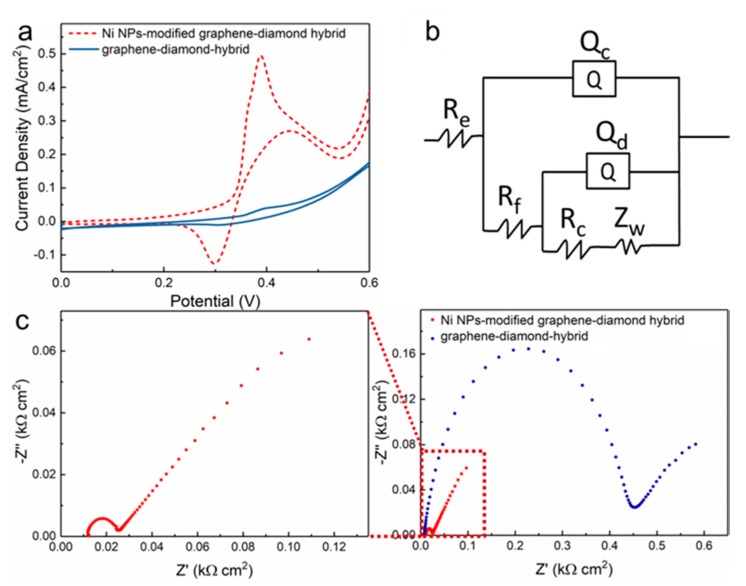
(**a**) Cyclic voltammetry (CV) responses of the graphene–diamond hybrid electrodes with and without Ni NPs modification in 0.5 M NaOH electrolyte containing 5 mM glucose at a scan rate of 20 mV s^−1^. (**b**) An equivalent circuit and (**c**) Nyquist plots of the graphene–diamond hybrid electrodes with and without Ni NPs modification with a frequency between 0.01–100,000 Hz and amplitude of 10 mV.

The Ni NPs-modified graphene–diamond hybrids were further fabricated into electrodes for electrochemical experiments, and for comparison, a graphene–diamond hybrid without decoration of Ni NPs was also prepared by immersing the sample in an etching solution (2 g CuSO_4_ and 10 mL HCl in 10 mL DI water) [40]. Based on the cyclic voltammetry (CV) method, Figure 3a shows the electrocatalytic performance of graphene–diamond hybrid electrodes with and without Ni NPs modification in 0.5 M NaOH electrolyte containing 5 mM glucose. Obviously, the peak current density of the Ni NPs-modified graphene–diamond hybrid shows a remarkable increase with a 20-fold improvement compared to the graphene–diamond hybrid without Ni NPs decoration. Such significant enhancement can be explained as follows [41]: (1) Ni NPs provide additional active sites during the electrocatalytic process and (2) the covalent bonding between Ni NPs and the graphene–diamond hybrid surface reduces the interfacial charge transfer resistance. The Nyquist plots of the graphene–diamond hybrid electrodes with and without Ni NPs modification in 1 M KCl solution containing 5 mM K_3_[Fe(CN)_6_] and 5 mM K_4_[Fe(CN)_6_] are presented in Figure 3c, and in Figure 3b, the corresponding fitting equivalent circuit is applied to analyze the measured impedance data. The fitting parameters are listed in Table 1: R_e_, R_f_, and R_c_ represent the resistance of electrolyte, the film electrode, and the charge transfer, respectively. Constant phase elements Q_c_ and Q_d_ represent the capacitance of the film electrode and the double layer. In the low frequency region, the Warburg resistance is represented by Z_w_. The marked decrease of the R_c_ of Ni NPs-modified graphene–diamond hybrid (0.6 Ω cm^2^, as without decoration: 353.8 Ω cm^2^) may be credited to the enhanced charge transfer, which means that the better interface between Ni NPs and the graphene–diamond hybrid due to the proposed in situ formation process, leading to the acceleration of the charge transfer rate [42]. In addition, the R_f_ of our sample (6.2 Ω cm^2^) is lower than that of the undecorated graphene–diamond hybrid (430.4 Ω cm^2^), because of the enhancement of electrical conductivity of the electrodes with Ni NPs modification [43]. 

**Figure 4:**
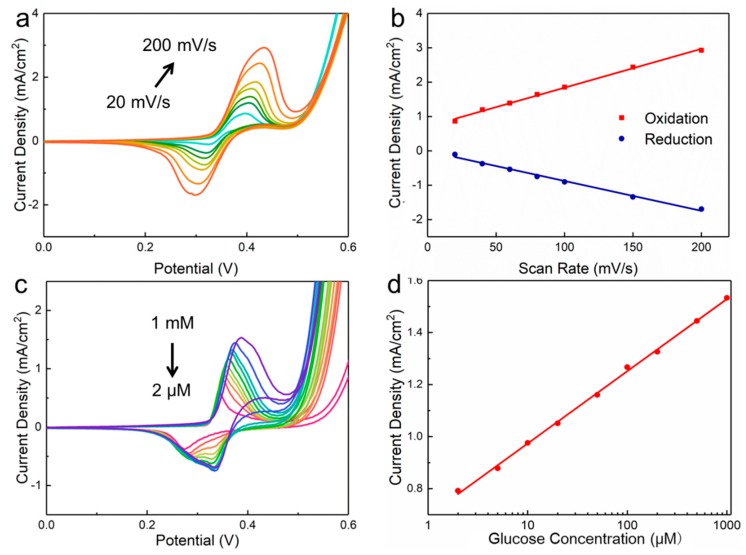
(**a**) CV responses of Ni NPs-modified graphene–diamond hybrid electrodes for glucose detection as a function of various scan rates (from inner to outer curves): 20, 40, 60, 80, 100, 150, and 200 mV s^−1^ in 0.5 M NaOH electrolyte containing 5 mM glucose and (**b**) the corresponding current density of oxidation and reduction peaks. (**c**) CV responses of Ni NPs-modified samples in 0.5 M NaOH electrolyte containing different glucose concentrations at a scan rate of 100 mV s^−1^. (**d**) Current density of devices as a function of glucose concentration.

The sensing performance of Ni NPs-modified graphene–diamond hybrid electrodes for detecting glucose was investigated by changing the scan rate of CV measurement, and the results are shown in Figure 4a. A pair of redox peaks can be observed at the potential range between 0–0.6 V. As reported [41], the two peaks originate from the redox reaction of Ni^3+^/Ni^2+^ couple, the reaction equations of which can be described as follows:

Ni + 2OH^−^ → Ni(OH)_2_ + 2e^−^ (1)

Ni(OH)_2_ + OH^−^ → NiOOH + H_2_O + e^−^ (2)

NiOOH + glucose → Ni(OH)_2_ + gluconolactone (3)

With an increase of scan rate, the anodic and cathodic peak currents increase monotonically, simultaneously accompanying the positive shift of the oxidation peak and the negative shift of the reduction peak, resulting in an increase of the peak separation because a higher over-potential needs to be applied to achieve the same electron transfer rate [44]. As displayed in Figure 4b, the current density of oxidation and reduction peaks as a function of the scan rate has a high correlation coefficient of 0.996 and 0.991, respectively, suggesting a surface-controlled process for glucose detection. Figure 4c presents the CV responses of Ni NPs-modified graphene–diamond hybrid electrodes in 0.5 M NaOH electrolyte containing different glucose concentrations at 100 mV s^−1^. The peak current density of our glucose sensors linearly increases with the increase of glucose concentration, and the oxidation peak shows a right shift when glucose concentration increases, which can be attributed to the limited diffusion of glucose on the surface of the electrode [45]. In Figure 4d, the linear fitting curve can be expressed as: I (mA) = 0.2777 log C (μM) + 0.6960 with a high correlation coefficient of 0.998. As a result, the low detection limit (LOD) of our glucose sensors is 2 μM, determined using the expression: LOD = 3s_b_/S, where S is the slope of the linear plot and s_b_ is the blank measurement in Figure 4d. 

A comparison of our sample with other Ni NPs-modified carbon-based electrodes is presented in Table 2, indicating the improved performance of our hybrid electrode in glucose detection [45,46,47,48]. Except Ni NPs, other nanomaterials such as cobalt phthalocyanine and CuO nanoflakes have also been used for sensing glucose and exhibit good electrochemical activity [49,50]. In our sensor the graphene layers play a role as electrical conducting films, and the good interface between the Ni nanoparticles and graphene guarantees efficient charge transfer during electrochemical detection, compared with pristine graphene–diamond. The improved sensing performance can be attributed to the high electrocatalytic activity of Ni NPs, and compared with the work reported by Wang [48], the interface between Ni NPs and graphene presents better performance than the interface of Ni NPs/graphene nanosheets/GCE, which results in a wider linear range of detection.

**Figure 5:**
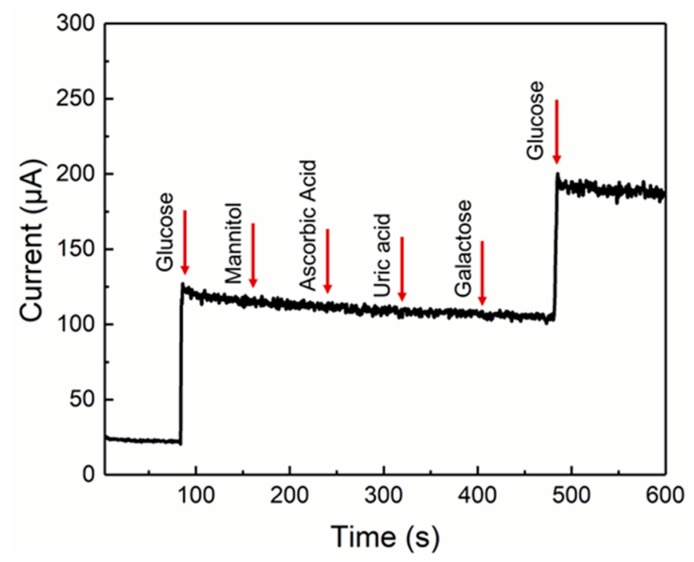
Non-enzymatic amperometric responses for the detection of glucose and interfering substances.

The selectivity of the sensor is quite significant for non-enzymatic sensors because many interfering substances with a higher electron transfer rate are easily oxidized, resulting in an interfering oxidation current for glucose detection. In our study, various interfering substances such as uric acid, ascorbic acid, and sugars (galactose, mannitol) (each 0.1 mM) were used to examine the selectivity of our glucose sensors using an amperometric method. As the results show in Figure 5, our Ni NPs-modified graphene–diamond hybrid electrode has good glucose specificity against other interfering substances, including sugars. 

## 4. Conclusions

In this study, we proposed a single-step method to fabricate Ni NPs-modified graphene–diamond hybrid electrodes for electrochemical glucose detection. Based on the sp^3^-to-sp^2^ conversion process, few-layer graphene decorated with Ni NPs could be formed simultaneously on the diamond surface. Compared to the undecorated sample, we demonstrated that Ni NPs-modified graphene–diamond hybrid electrodes have superior electrocatalytic performance in glucose detection, showing a linear dynamic range of between 2 μM–1 mM and a low detection limit of 2 μM. The improved sensing performance can be attributed to the high electrocatalytic activity of Ni NPs, as well as a good interface between Ni NPs and graphene based on our single-step formation process. The glucose sensors have the potential for efficient monitoring of blood glucose levels or other diabetic conditions.

## Figures and Tables

**Table 1:** The fitting parameters of the fitting equivalent circuit.

	R_e_ (ohm)	Q_c_ (F)	n_c_	R_f_ (ohm)	Q_d_ (F)	n_d_	R_c_ (ohm)	Z_w_ (ohm^−1^·s^0.5^/cm^2^)
Ni NPs-modified graphene–diamond hybrid	194.8	1.3 × 10^−6^	0.90	103.7	7.6 × 10^−5^	0.8	11.36	0.002
Graphene–diamond hybrid	127.7	7.3 × 10^−7^	0.84	7173	1.0 × 10^−3^	0.54	5896	7 × 10^−4^

**Table 2:** Glucose sensing performance using Ni NPs-modified carbon-based electrodes.

	Linear Range (mM)	Low Detection Limit (μM)	Ref.
Ni NPs/Boron doped diamond	0.1–10	2.7	[46]
NiCoO/Carbon nanotube	0.01–12.12	6.0	[47]
Ni NPs/Graphene nanosheets	0.005–0.55	1.9	[48]
Ni NPs-modified graphene–diamond	0.002–1	2.0	This work
